# Expert Consensus on Self-Reported Physical, Nutritional, and Psychological Screening Tools for Prehabilitation in Gastrointestinal Cancer Surgery: An International Delphi Study

**DOI:** 10.1245/s10434-026-19356-z

**Published:** 2026-03-14

**Authors:** Jack Reeves, Sascha Karunaratne, Kate Alexander, Alexandria Petridis, Sharon Carey, Haryana M. Dhillon, Linda Denehy, Michael Solomon, Bernhard Riedel, Cherry Koh, Kate McBride, Chelsia Gillis, Kate White, Nicholas Hirst, Zaid Shadid, Ishraque Rahman, Neil Pillinger, Daniel Steffens, Global (P)rehabilitation Initiative Collaborative Group, Abdalla Hadhoud, Abdelatte Ghanam, Abdelilah Ghannam, Abdelmoiz Mohamed, Abdelrahim Ahmed, Abdelrahman Mohamedali, Abdirahman Osman, Abdulaleem A. Esmail, Abdulhamid Shaban, Abdulrahman Mohammed, Abdulzahra Hussain, Abdullah Al-Mehdhar, Abdullah Alyami, Achraf Drissi-El-Bouzaidi, Aeshah Alqathafi, Ahmed Ahmayda, Ahmed Elsayed Tharwat Abdelsalam, Ahmed Ibrahim MohamedMohamed, Ahmed Musbah, Ahmed Shoman, Ahmed Wahaballah, Aiman Al-Touny, Aiman Hussien, Akrem Amer, Amina Houmada, Amr Badawy, Andrea B. Maier, Andreas Denys, Andrew Davies, Angel Becerra-Bolaños, Angela Jerath, Anjum Khan Joad, Anthony Absalom, Areej Ahmed Al-Qaidani, Aras Emre Canda, Ashleigh Maske, Ashu Sara Mathai, Awab Hamid, Ayah Attallah, Ayten Saracoglu, Badr Aldeen Al-Tayar, Barbara Sinner, Basantha Kumar Rayani, Behic Girgin, Bernhard Riedel, Bisher Sawaf, Birgitte Brandstrup, Bodil Andersson, Brendan Moran, Brendon Roxburgh, Bruno Romanò, Carol Keen, Catarina Tiselius, Chelsia Gillis, Chloe Grimmett, Christian Beilstein, Cindy Yeoh, Claus Anders Bertelsen, Dario Baratti, Darren Au, David Clark, Declan Dunne, Denny Levett, Despoina Liotiri, Dimitrios Schizas, Dominique Engel, Dorian Krsul, Edoardo Virgilio, Ebrahim Ali Mohammed Amrein, Eleni Kitsou, Elharith Abduelfattah Gaber Mohammed, Elissavet Anestiadou, Emily Traer, Engy Elkoury, Enrique Alday Muñoz, Erik Nordenström, Erdinç Kamer, Eslam Elshenawy, Ewen Harrison, Fabio Ausania, Fadwa Fuad Abdulwahid Mansoor, Fatima Elmobark, Fernando Arduini, Fevzi Cengiz, Francesk Mulita, Francesco Mongelli, Francesco Pata, Frank Frizelle, Frederik Berrevoet, Gabrielle H. Van Ramshorst, Gianluca Pellino, Grainne Sheill, Gregg Nelson, Hadeer Alsadeg Mohammed, Hande Gurbuz, Hans D. De Boer, Hamid Aslami, Hamza Elhamzaoui, Hashem Abu Serhan, Haidara Bohsas, Heleen Driessens, Helena Pereira, Helmut Raab, Iacopo Cappellini, Ian Randall, Ianthe Boden, Ileana Lulic, Ilenia Merlini, Ina Ismiarti Shariffuddin, Jakub Chmelo, James Durrand, Janine Bothe, Jason Park, Jayson Moloney, Jenelle Loeliger, Jonathan Hardman, Joost Klaase, Jose Ignacio García-Sánchez, Julia Dubowitz, Julie Hallet, Junichiro Kawamura, Jyotirmay Kirtania, Kari Clifford, Kariem El-Boghdadly, Kate McBride, Khaled Abdelwahab, Khalid Osman Mohamed, Kheng-Seong Ng, Khloud Ali M. Dahan Saeed, Kimberley Bostock, Kilian Brown, Kristy-Lee Raso, Kyrillos Nassim, Kylie Hill, Kwong Weng Loh, Lara Edbrooke, Laura Lorenzon, Lewis Matthews, Linda A. Antonucci, Linda Edgar, Lissa Spencer, Louise Brennan, Magdalena Sielewicz, Magdalena Taube, Mahmoud Eissa, Majd Chaaban, Malcolm West, Malak Ghemmeid, Malek Balkhi, Manazir Athar, Marc Pocard, Marc Van De Velde, Marco Sparavigna, Marcos Gómez Ruiz, Marie-Louise Lydrup, María García Nebreda, Marta Ubre, Massimo Falconi, Michael Hii, Michael Kelly, Miguel Leon-Arellano, Moataz Maher Emara, Mohamed Al Gharyani, Mohamed Alsori, Mohamed Elbahnasawy, Mohamed Fouad Abdrabo, Mohamed Ghula, Mohamed Said, Mohamed Sherif Ali Ahmed, Mohammed Abdallah Mohammed Kheer Almotaared, Mohammed Al-Shehari, Mohammed Anass Majbar, Mohammed Izzaldin, Mohammed Omar, Mohammed Sarhan Alkhallatee, Mogahid Ahmed, Momin Hilles, Mostafa Algabri, Moyasar Awad, Msara Haider, Muhamad Kanjo, Nada Wd Badr, Nader Hanna, Nassila Krita, Natasa Kovac, Neil Pillinger, Nermina Rizvanovic, Nivedhyaa Srinivasaraghavan, Nurhilal Kiziltoprak, Orestis Ioannidis, Omar Ahmed Abdelwahab, Osman Osman, Oumayma Lahnaoui, Pankaj Roy, Pamela Klassen, Patrice Forget, Patricia Sylla, Paul Sutton, Pawel Kabata, Pawel Mroczkowski, Per J. Nilsson, Petra Bor, Peter Ihnát, Peter Noordzij, Peter Paal, Philip H. Pucher, Piotr Major, Qiang Wang, Rafat Muharam, Raquel García Álvarez, Rasha Shrayyef, Rashed Alweshah, Reham Atef Zaki Mohamed, Rhonda Farrell, Riccardo Caruso, Riyadh Ikreedeegh, Robert Arnold, Roberto Falz, Rohan Miegel, Rosalia Navarro-Perez, Ross Dolan, Safia Adem, Saleem Ahmed, Sami Sannoufa, Sandra Auld, Sara S. Elsheikh, Sarah Abdelmohsen, Sarah Braungart, Sasa Rajsic, Sawsan Ismail, Semra Demirli Atici, Seymanur Altintas Filizoglu, Shannon Philp, Sharon Carey, Shelby Yaceczko, Simone Manfredelli, Sorrel Burden, Sophie Hogan, Stefano Mancin, Styliani Apostolaki, Susana González-Suárez, Talat Abu Salem, Tania Triantafyllou, Tarik Sammour, Tim Bright, Tom Parkington, Varsha Gorey, Vasileios Mousafeiris, Varut Lohsiriwat, Victor Zaydfudim, Walter R. Frontera, Walid Ibrahim, Waled Aljbali, Wilton Van Klei, Yasemin Yıldırıms, Yousef Abo Alsoud, Zakaria Belkhadir, Abdurrahman Haddud, Abubkr Elbushary, Ahmed Mohieldin Ahmed Abdalla, Alessio Giordano, Alexander Heriot, Alexander W. Phillips, Alexey Ovezov, Alfredo Annicchiarico, Algazali Jadalla, Ali Elhadidy, Alice Jay, Alison Jackson, Almu‘atasim Khamees, Amanh Hussien, Amine Souadka, Amr Al Hammoud, André Torbey, Anton Burlaka, Aurélien Venara, Bashir Albakosh, Brahim El Ahamdi, Cillian Clancy, Claire Hackett, Cristina Petrisor, Daniel Santa Mina, Evgenia Mela, Hajer Alsadeg Mohammed, Ian Daniels, Julio Mayol, June Davis, Kelcie Cole, Kemal Tolga Saracoglu, Ker-Kan Tan, Khabab Abbasher Hussien Mohamed Ahmed, Liane Feldman, Mohamed Attia Elfadali, Mohammad Badr Almoshantaf, Mohammed Babikir, Moise Alida, Osama Mohamed, Padraig Buggy, Pierre-grégoire Guinot, Ragdah Mohammed, Rajeswari Alagirimunuswamy, Rand AlShrbaji, Rayan Wedatalla, Selman Sökmen, Shady Abdo Saad Ramadan, Sohaib Hasan Abdullah Ezzi, Soumia Bechka, Stephanie Castagnini, Wafaa Abdel-Elsalam

**Affiliations:** 1 Surgical Outcomes Research Centre (SOuRCe), Royal Prince Alfred Hospital, Sydney, Australia; 2 Discipline of Physiotherapy, Graduate School of Health, Faculty of Health, University of Technology Sydney, Sydney, NSW Australia; 3 Physiotherapy Department, Royal Prince Alfred Hospital, Sydney, Australia; 4 Faculty of Medicine and Health, Central Clinical School, The University of Sydney, Sydney, Australia; 5 Institute of Academic Surgery (IAS), Royal Prince Alfred Hospital, Sydney, Australia; 6 Faculty of Science, School of Psychology, Psycho-oncology Cooperative Research Group, The University of Sydney, Sydney, Australia; 7 Department of Health Services Research, Allied Health, Peter MacCallum Cancer Centre, Melbourne, Australia; 8 Department of Physiotherapy, Faculty of Medicine Dentistry and Health Sciences, The University of Melbourne, Melbourne, Australia; 9 The Sir Peter MacCallum Department of Oncology, and The Department of Critical Care, The University of Melbourne, Melbourne, Australia; 10 Department of Anaesthetics, Perioperative Medicine, and Pain Medicine, Peter MacCallum Cancer Centre, Melbourne, Australia; 11 School of Human Nutrition, McGill University, Montreal, QC Canada; 12 Susan Wakil School of Nursing, Faculty of Medicine and Health and Daffodil Centre, The University of Sydney, Sydney, Australia; 13 Department of Anaesthetics, Royal Prince Alfred Hospital, Sydney, Australia

**Keywords:** Delphi study, Prehabilitation, Preoperative screening, Gastrointestinal cancer

## Abstract

**Background:**

Prehabilitation can decrease postoperative complications and enhance recovery for people with gastrointestinal cancer. Preoperative screening may identify individuals at highest risk of poor postoperative outcomes, enabling targeted and tailored interventions. Despite this, optimal tools for screening patients before surgery remain unclear. This Delphi study sought to achieve international consensus on appropriate screening tools to identify patients at increased risk of postoperative complications before undergoing gastrointestinal cancer surgery.

**Methods:**

A three-round iterative Delphi survey was distributed to a global multidisciplinary network of prehabilitation experts. Respondents were asked to rate screening tools, identified through a scoping review, on a 5-point Likert scale based on the appropriateness of their use for adult patients undergoing gastrointestinal cancer surgery. The screening tools were categorized as evaluating either physical, nutritional, or psychological domains. A consensus criterion was applied in the second and third rounds, which required ≥ 70% of respondents to rate a given tool as ≥ 4 for it to proceed to future rounds.

**Results:**

Overall, 308 self-nominated prehabilitation experts rated the screening tools. Of 26 screening tools (including 9 physical, 7 nutritional, and 10 psychological), consensus was achieved for three physical, three nutritional, and four psychological tools.

**Conclusion:**

Based on Delphi consensus of global prehabilitation experts, 10 self-reported screening tools reached consensus. Given that tool selection was informed by Delphi consensus rather than empirical validation, prospective studies are necessary to assess whether these instruments accurately stratify surgical risk and identify candidates most likely to benefit from prehabilitation.

**Supplementary Information:**

The online version contains supplementary material available at https://doi.org/10.1245/s10434-026-19356-z.

Cancer remains a leading cause of mortality worldwide, accounting for an estimated 9.7 million deaths per year.^1^ Gastrointestinal (GI) cancers have shown rising incidence, driven by an ageing global population, currently accounting for one third of all cancer-related deaths.^2^

Surgery is the primary intervention for patients with GI cancer, either with or without neoadjuvant therapy such as radiation or systemic cancer therapy. Many individuals undergoing GI cancer surgery experience postoperative complications, which can lead to longer hospital stays, higher hospital readmission rates, reduced timely adjuvant therapy, poorer long-term quality-of-life outcomes, and increased health care costs^3,4^ Risk factors for postoperative complications have previously been identified,^5–9^ many of which are modifiable through preoperative care such as prehabilitation initiatives to optimize physical, nutritional, and psychological domains.

A scoping review of randomized trials defined prehabilitation as “a process from diagnosis to surgery comprising one or more preoperative interventions of exercise, nutrition, psychological strategies, and respiratory training that aim to enhance functional capacity and physiologic reserve to allow patients to withstand surgical stressors, improve postoperative outcomes, and facilitate recovery.”(p310).^10^

Despite evidence from randomized controlled trials (RCTs) ^11–13^ supporting surgical prehabilitation as an effective strategy to enhance patient outcomes and reduce postoperative complications, prehabilitation is not routinely implemented in many health care settings, often due to resource limitations.^14–17^ Given this, there is a significant role for preoperative screening to identify high-risk individuals most likely to benefit, allowing for targeted interventions.

Several tools exist to evaluate the preoperative health of surgical candidates within physical, nutritional, and psychological domains, but none address all three. Whereas some tools require physical assessment, inclusion of biochemical markers, or clinician administration, those that are patient-reported allow for timely and patient-centered assessment, especially when delivered electronically.^18^ This is important because prehabilitation interventions resulting from screening need to occur between diagnosis and surgery, a period that often can be short given its independent association with overall survival.^19^ In fact, a recent editorial advocated for transforming surgical waiting lists into preparation opportunities to encourage active engagement by patients and health care providers.^20^

This study formed part of a broader research program investigating preoperative screening tools for patients undergoing gastrointestinal cancer surgery with a published protocol.^21^ The overarching aim was to develop, validate, and implement a simple, online, self-reported preoperative risk-stratification tool to identify modifiable physical, nutritional, and psychological factors enabling targeted and tailored prehabilitation interventions.^21^ This Delphi consensus study followed a scoping review aimed at identifying the landscape of preoperative screening tools currently used in gastrointestinal cancer cohorts. The aim of this study was to achieve international consensus among prehabilitation experts on the most appropriate tri-modal (physical, nutritional, psychological) screening tools for patients undergoing gastrointestinal cancer surgery.

## Methods

### Study Design and Setting

Delphi methodology was implemented according to research guidelines^22^ to gain international consensus on the most appropriate preoperative patient-reported screening tools to assess physical, nutritional, and psychological domains for patients undergoing gastrointestinal cancer surgery. A three-round iterative survey was used to rate screening tools identified through a scoping review. A protocol for this Delphi study and the preceding scoping review have been published previously.^21^

The Delphi survey was distributed via a secure data management software, Research Electronic Data Capture (REDCap; Vanderbilt University, Nashville, TN, USA)^23^ between June and October 2025. The study adhered to the Conducting and Reporting of Delphi Studies (CREDES) guidelines for reporting Delphi studies.^24^ Ethical approval was obtained from the Sydney Local Health District (SLHD) Human Research and Ethics Committee (X25-0008/ETH00137).

### Participants

A multidisciplinary panel of international experts in prehabilitation were invited to take part in the study. The experts were approached via e-mail through the Global Prehabilitation Initiative (a group of > 3000 self-nominated prehabilitation experts across 6 continents). Authors were identified in literature through the preceding scoping review, and the invitation was disseminated through networks of experts. Consent for participation was implied for those who responded to the Delphi invitation and completed the first-round survey. To be eligible, participants needed to be ≥ 18 years of age, be able to respond to the survey in English, and be self-confirmed as a clinician or academic with expertise in prehabilitation or preoperative screening.

### First-Round Survey

In the first round, the respondents rated the appropriateness of the self-reported preoperative screening tools for gastrointestinal cancer surgery patients using a 5-point Likert scale as follows: 1 (no importance), 2 (limited importance), 3 (neither important nor unimportant), 4 (somewhat important), and 5 (critically important). The screening tools were categorized as physical, nutritional, or psychological. To assist in rating the tools, the responders were provided with a copy of the screening tool as well as information about the tool including the tool name, a description of the tool including its intended use, the number of items, the expected time to complete, the domains within the tool, the response format (i.e., multiple-choice, yes/no, Likert scale), the recall period, and how the tool is scored. The responders were asked to vary their responses within the survey as a help to avoid response-set bias through non-differentiation, enabling researchers to discriminate between tools for following rounds (Supplementary Document 1).

The following criteria, based on the criteria used from a previous Delphi study, were applied to determine whether a consensus had been achieved on the appropriateness of each tool:^25^

- *Consensus in:* ≥ 70% of responders marked the tool as important (≥ 4).
- *Consensus out*: ≥ 70% of responders marked the tool as of limited importance (≤ 2).
- *No consensus*: neither of the first two criteria are met.

Additionally, the responders were to identify any other tool relevant to preoperative screening for patients undergoing gastrointestinal cancer surgery. The first-round survey also collected demographic information (age, gender, highest degree, country of origin, profession, cancer type).

### Second-Round Survey

Based on feedback from the first-round survey, the respondents in the second round selected specific domains to rate (i.e., participants identified that their expertise was confined to a particular domain, e.g., exercise but not psychology). Round two included all preoperative screening tools that met the criteria for “consensus in” or “no consensus” as well as additional tools suggested by the first-round responders, which were patient-reported and available to researchers. Screening tools meeting the criteria for “consensus out” were not included in the second round.

The second-round survey was sent via email to all the participants of the first round. Responders were asked to rate the refined list of preoperative screening tools, varying their ratings to discriminate between tools. The same consensus criteria were used to assess scores in the second round. The respondents were provided with their scores from the first-round survey, as well as the mean score of all the responders for each screening tool included in the second round.

### Third-Round Survey

The third-round survey included all the preoperative screening tools meeting the criteria for “consensus in” in the second-round survey. The responders were again asked to rate the further refined list of screening tools. After the third-round survey, final consensus was achieved if a tool met the “consensus in” criteria.

### Data Analysis

The data for each survey round are characterized using descriptive statistics. For all the Delphi rounds, the data are displayed with frequency (percentage) and mean (standard deviation). Survey responses with partial answers were included in the analysis.

## Results

**Fig. 1:**
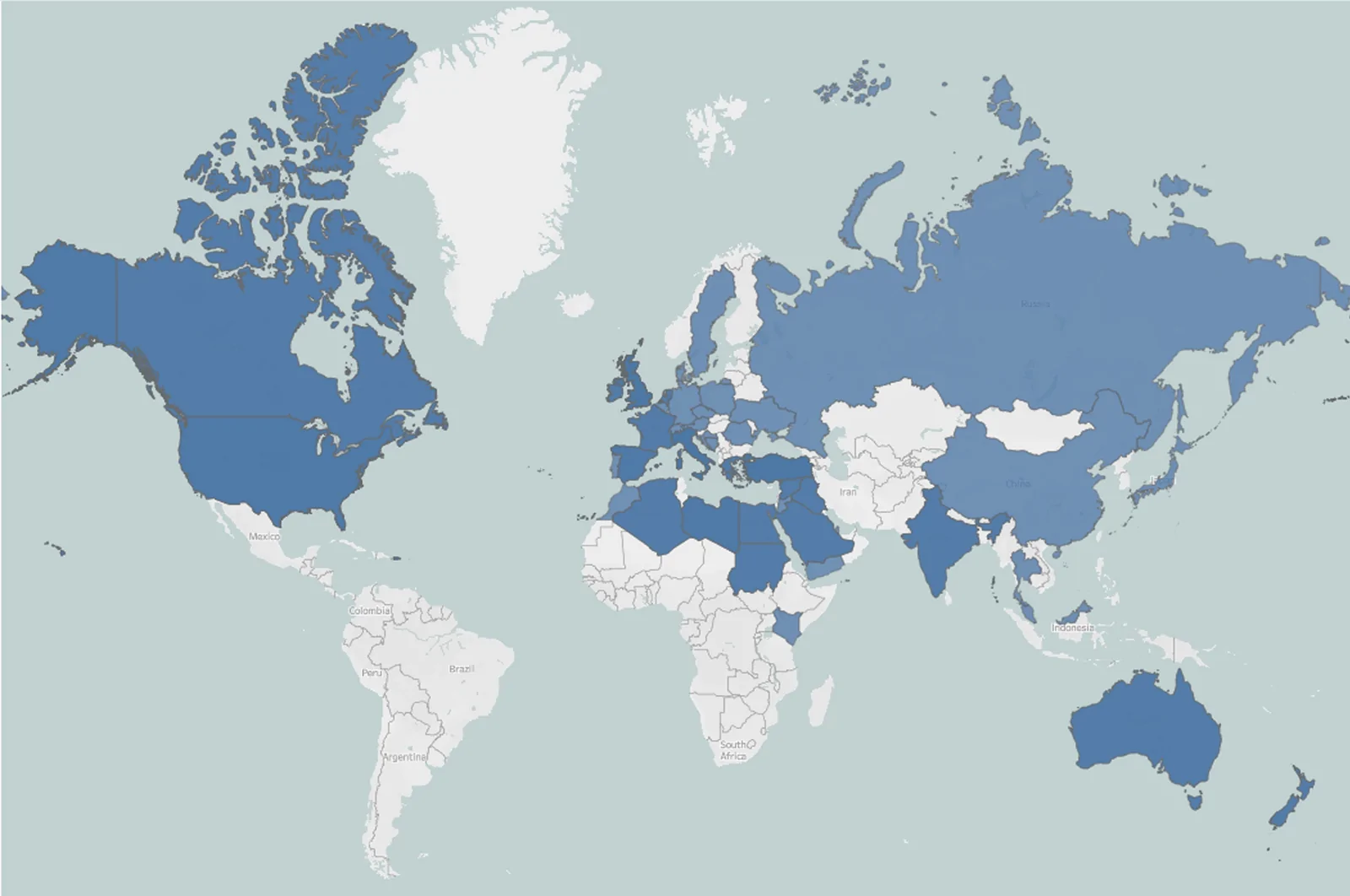
Global distribution of prehabilitation experts who responded to the Delphi survey. Blue indicates countries in which respondent(s) reside

A total of 308 participants responded to the Delphi surveys. Demographic information on the respondents is reported in Table 1. All 308 respondents (100%) completed the first-round survey, with 231 (75%) completing the second round, and 230 (75%) completing the third round. Prehabilitation experts from 49 countries responded, with global distribution demonstrated in Fig. 1. Of the 308 respondents, 67 (22%) reported prehabilitation as the standard of care for all the patients at their center, and 164 (54%) reported prehabilitation as the standard of care for a select group of patients at their center. The majority of the respondents were surgeons (*n* = 129, 43%) or anesthesiologists (*n* = 69, 23%).

**Table 1:** Demographic information of prehabilitation experts who responded to the Delphi survey (*n* = 308)

Characteristics	Participants *n* (%)
*Age (years)*	
< 31	69 (23)
31–40	79 (26)
41–50	96 (32)
51–60	41 (14)
61–70	14 (5)
> 70	2 ( < 1)
*Gender*	
Male	196 (65)
*Profession*	
Surgeon	129 (43)
Anesthetist	69 (23)
Other	42 (14)
Academic	24 (8)
Dietitian	15 (5)
Physiotherapist	11 (4)
Nurse	7 (2)
Psychiatrist	3 (1)
Psychologist	1 ( < 1)
*Highest education*	
Doctoral degree	84 (28)
Professional doctorate	88 (29)
Master’s degree	46 (15)
Bachelor honors degree	16 (5)
Bachelor’s degree	56 (19)
Other	11 (4)
*No. of respondents reporting each cancer type at their center*	
Gastrointestinal	275
Gyaecologic	143
Urologic	133
Head and neck	115
Thoracic	92
Neurologic	86
Other	29

Data expressed as frequency (%) or mean (SD)

**Fig. 2:**
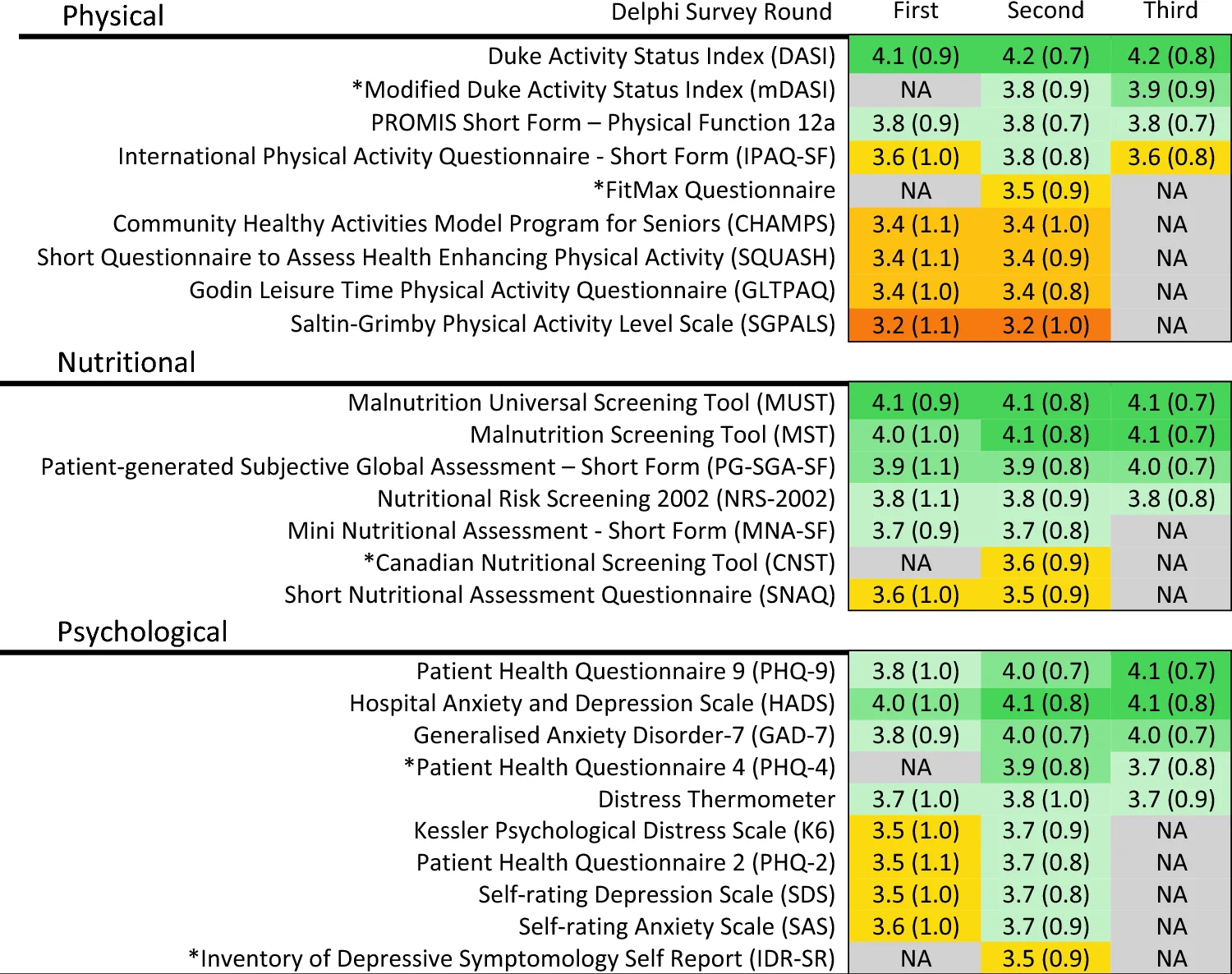
Mean (SD) screening tool scores using a 5-point Likert scale by prehabilitation experts for each Delphi round. *Tools that met the “consensus in” criteria in the final Delphi rounds

The results for all three Delphi rounds, with consensus criteria applied, are reported in Table 2. The mean scores and standard deviations, as voted by the respondents, are shown for each Delphi round in Fig. 2.

**Table 2:** Delphi survey results based on consensus criteria

Tool name	First-round survey	Second-round survey	Third-round survey
	(*n* = 308/100%)	(*n* = 231/75%)	(*n* = 230/75%)
	Rated as ≥4/total responses (%)		
*Physical*			
Duke activity status index (DASI)	**259/306 (85)**	**166/182 (91)**	**163/185 (88)**
Modified duke activity status index (mDASI)^a^	NA	**133/183 (73)**	**136/185 (74)**
PROMIS short form–physical function 12a	**220/305 (72)**	**137/181 (76)**	**133/185 (72)**
International physical activity questionnaire–short form (IPAQ-SF)	192/305 (63)	**126/181 (70)**	115/185 (62)
FitMax questionnaire^a^	NA	107/184 (58)	NA
Community healthy activities model program for seniors (CHAMPS)	170/307 (55)	94/181 (52)	NA
Short questionnaire to assess health enhancing physical activity (SQUASH)	155/305 (51)	87/178 (49)	NA
Godin leisure time physical activity questionnaire (GLTPAQ)	158/305 (52)	81/179 (45)	NA
Saltin-grimby physical activity level scale (SGPALS)	136/304 (45)	69/182 (38)	NA
*Nutritional*			
Malnutrition universal screening tool (MUST)	**252/305 (83)**	**135/163 (83)**	**150/171 (88)**
Malnutrition screening tool (MST)	**236/305 (77)**	**139/163 (85)**	**149/171 (87)**
Patient-generated subjective global assessment–short form (PG-SGA-SF)	**214/304 (70)**	**125/163 (77)**	**127/171 (74)**
Nutritional risk screening 2002 (NRS-2002)	202/305 (66)	**120/163 (74)**	111/171 (65)
Mini nutritional assessment–short form (MNA-SF)	192/305 (63)	112/163 (69)	NA
Canadian nutritional screening tool (CNST)^a^	NA	97/163 (60)	NA
Short nutritional assessment questionnaire (SNAQ)	176/304 (58)	95/163 (58)	NA
*Psychological*			
Patient health questionnaire-9 (PHQ-9)	207/305 (68)	**94/116 (81)**	**102/116 (88)**
Hospital anxiety and depression scale (HADS)	**231/305 (76)**	**101/116 (87)**	**99/116 (85)**
Generalised anxiety disorder-7 (GAD-7)	**213/305 (70)**	**94/116 (81)**	**98/116 (84)**
Patient health questionnaire-4 (PHQ-4)^a^	NA	**85/116 (73)**	**81/116 (70)**
Distress thermometer	201/304 (66)	**87/116 (75)**	74/116 (64)
Kessler psychological distress scale (K6)	166/305 (54)	73/115 (63)	NA
Patient health questionnaire-2 (PHQ-2)	169/305 (55)	72/116 (62)	NA
Self-rating depression scale (SDS)	178/305 (58)	72/116 (62)	NA
Self-rating anxiety Scale (SAS)	180/305 (59)	70/116 (60)	NA
Inventory of depressive symptomology self report (IDR-SR)^a^	NA	62/116 (55)	NA

SD, standard deviation; NA (first round), not applicable due to not being listed in round 1 but subsequently being added due to round 1 respondent suggestions; NA (third round), not applicable due to being screened out in the second round due to not meeting the “consensus in” criteria of  ≥ 70% of responders marking the tool as important ( ≥ 4) in round 2 of the Delphi process

^a^Denotes tools recommended by prehabilitation experts during the first-round survey. Bold values represent tools that met the “consensus in” criteria (applicable to second- and third-round surveys). “Consensus in” criteria = tools rated as somewhat important (4) or critically important (5) on a 5-point Likert scale

### First-Round Survey

In the first round, the respondents rated the appropriateness of seven physical, six nutritional, and eight psychological tools. Once consensus criteria were applied, two physical (29%), three nutritional (50%), and two psychological (22%) tools met consensus. The respondents identified two physical, one nutritional, and two psychological additional self-reported screening tools to be included in the second-round survey.

### Second-Round Survey

In the second round, all tools (including newly identified tools) were included because no tools met the “consensus out” criteria during the first round. In the second round, once the consensus criteria were applied, four physical (44%), four nutritional (57%), and five psychological (50%) tools that met the “consensus in” criteria. Only tools that met the “consensus in” criteria were included in the third-round survey.

### Third-Round Survey

In the final round, three physical (75%), three nutritional (75%), and four psychological (80%) tools met consensus as appropriate screening tools for adults undergoing gastrointestinal cancer surgery. The physical tools included the Duke Activity Status Index (DASI),^26^ the Modified Duke Activity Status Index (mDASI),^27^ and the Patient-Reported Outcome Measurement System (PROMIS) Short Form–Physical Function 12a.^28^ The nutritional tools included the Malnutrition Universal Screening Tool (MUST).^29^ the Malnutrition Screening Tool (MST),^30^ and the Patient-Generated Subjective Global Assessment–Short Form (PG-SGA-SF).^31^ The psychological tools included the Patient Health Questionnaire-9 (PHQ-9),^32^ the Patient Health Questionnaire-4 (PHQ-4),^33^ the Hospital Anxiety and Depression Scale (HADS),^34^ and the Generalised Anxiety Disorder-7 (GAD-7).^35^ Screening tool properties can be seen in Table 3.

**Table 3:** Properties of screening tools that met “consensus in” in the final Delphi round

Screening tool	Domain	No. of items	Recall period	Response format	Time to complete (min)	Freely available^a^
Duke activity status index (DASI)	Physical	12	Current	Yes/no checklist	< 5	N
Modified duke activity status index (mDASI)	Physical	5	Current	Yes/no checklist	< 2	N
PROMIS short form–physical function 12a	Physical	12	7 days	5-Point Likert scale	< 5	Y
Malnutrition universal Screening tool (MUST)	Nutritional	3	3–6 months	Multiple-choice	3–5	N
Malnutrition screening tool (MST)	Nutritional	2	6 months	Multiple-choice	< 2	Y
Patient-generated SGA short form (PG-SGA-SF)	Nutritional	4	1 month	Multiple-choice	< 5	N
Patient health questionnaire-9 (PHQ-9)	Psychological	9	2 weeks	4-Point Likert scale	< 2	Y
Patient health questionnaire-4 (PHQ-4)	Psychological	4	2 weeks	4-Point Likert scale	< 1	Y
Hospital anxiety and depression Scale (HADS)	Psychological	14	1 week	Multiple-choice	< 5	N
Generalised anxiety disorder-7 (GAD-7)	Psychological	7	2 weeks	4-Point Likert scale	1–2	Y

^a^“Freely available” in this context implies that the tool can be readily used without costs or permissions

## Discussion

This Delphi study identified three physical, three nutritional, and four psychological screening tools that met consensus as appropriate for those undergoing gastrointestinal cancer surgery through consensus of a global multidisciplinary panel of prehabilitation experts.

A previous Delphi study explored research priorities for prehabilitation of patients undergoing cancer surgery.^25^ The second highest rated research priority was to “identify populations most likely to benefit from prehabilitation.” Given the time and resource constraints in many settings, early identification of those people at greatest risk of postoperative complications offers the opportunity for effective targeted interventions. Screening tools are a simple and cost-effective way to identify those who might be at risk requiring further assessment.

### Physical Screening Tools

Three physical screening tools met consensus. The Duke Activity Status Index (DASI) was designed to assess the functional capacity of adults by evaluating an individual’s ability to perform daily activities, ranging from basic self-care to strenuous physical tasks. A cohort study assessing the predictive utility of the DASI for postoperative complications in 100 participants undergoing colorectal resection found that lower DASI scores were associated with higher odds of complications (odds ratio [OR], 1.08; 95% confidence interval [CI],1.03–1.14).^36^ This result corroborates a larger cohort study (*n* = 1546) assessing the DASI in a non-cardiac surgery population of participants at a higher cardiac risk. The study found that below a threshold of 34 points, participants had greater risk of 30-day mortality and 1-year disability.^37^ Evidence exists to support the DASI as a responsive tool with adequate construct validity specific to colorectal surgery.^38^

The modified Duke Activity Status Index (mDASI), which involves five (or four) of the original DASI questions, also met consensus.^27^ This screening tool was developed by evaluating cardiopulmonary exercise test data and DASI scores of 1455 trial participants who underwent non-cardiac surgery to provide a simplified measure able to identify people with adequate functional capacity compared with those who may benefit from prehabilitation interventions.

The Patient-Reported Outcome Measurement Information System (PROMIS) group has developed numerous outcomes tools including the Short Form–Physical Function, used to assess self-reported capacity to perform physical activities of daily living. Based on a scoping review evaluating use of screening tools by those undergoing gastrointestinal cancer surgery, the “12a” (a version of the tool) was identified in two studies evaluating home-based prehabilitation exercise programs for those with potentially resectable pancreatic cancer^39^^,^^40^ and met consensus among the respondents in the current Delphi study.

### Nutritional Screening Tools

The Patient-Generated Subjective Global Assessment Short Form (PG-SGA-SF) is another commonly used nutritional screening tool often used with cancer patients undergoing surgery. It is a patient-reported adaptation of the SGA (which involves a clinician assessment) and has previously been deemed as feasible as a patient-reported measure in an oncologic population.^41^ Based on receiver operating characteristic analysis in a study evaluating an ambulatory oncologic population (*n* = 300), a cut-point of ≥ 3 (area under the curve [AUC], 0.85) has been suggested.^31^

The Malnutrition Universal Screening Tool (MUST) is a five-step screening tool developed to identify adults who are malnourished or at risk of malnutrition. It assesses nutritional status based on body mass index (BMI), unintentional weight loss, and effect of acute disease, culminating in a risk score to guide management. A systematic review evaluated the MUST for effectiveness in predicting poor postoperative outcomes for those with colorectal cancer. Based on seven included studies (*n* = 1950), those rated by MUST as “high risk” versus “low risk” had a longer hospital stay (27 vs 14 days) and a higher prevalence of postoperative complications (41 vs. 9%).^42^ A briefer tool that met consensus was the Malnutrition Screening Tool (MST), a two-question tool developed to identify risk of malnutrition in adults in both hospital and ambulatory settings. Scores of ≥ 2 (total possible score 5) signify malnutrition. Although this tool met consensus as appropriate in the current Delphi study, it has poorer sensitivity (0.78; 95% CI, 0.64–0.88) and specificity (0.82; 95% CI, 0.76–0.87) than a reference standard of the SGA or PGA-SGA (both longer versions that include a clinician assessment section) based on a systematic review and meta-analyses.^43^

### Psychological Screening Tools

The Patient Health Questionnaire-9 (PHQ-9) is a self-administered tool designed to screen, diagnose, and monitor the severity of depression in individuals age 12 years or older. One consideration in implementing the PHQ-9 in a routine preoperative assessment is its item assessing suicidal ideation, which requires prompt follow-up evaluation by a trained health care professional. Although specific to a cancer population, a study has shown that only one third of respondents who report suicidal thoughts on the PHQ-9 continue to report suicidal ideation in subsequent follow-up evaluation.^44^ The PHQ-9 has been shown to perform well in identifying depression in cancer populations based on cutoff criteria for mild (5–9), moderate (10–14), and severe ( ≥ 15) depression.^45^

The Generalised Anxiety Disorder (GAD-7) is used to screen and measure anxiety severity in adults. Recently, a diagnostic cut-point of ≥ 8 points (21 total possible points) was recently suggested as optimal in oncologic populations.^46^ The Patient Health Questionnaire-4 (PHQ-4) also met consensus as an appropriate screening tool. This tool uses two items from the PHQ-9 and two items from the GAD-7 to provide a brief measure of both anxiety and depression in a single tool. Similarly, the widely used Hospital Anxiety and Depression Scale (HADS), which also met consensus, assesses both anxiety and depression. A previous systematic review and meta-analysis suggest that specific to cancer settings, the HADS may be poor at diagnosing cases of anxiety and depression, yet may be appropriate for the purpose of screening when its length (14 items) is considered.^47^

### Considerations

A total of 10 screening tools met expert consensus as appropriate for patients undergoing gastrointestinal cancer surgery. The choice of which tool to use should be based on the context and setting in which it is used. Clinicians, researchers, and policymakers need to consider aspects of each tool (e.g., cost, time to complete, recall period, and licensing costs) (Table 3) before deciding which tool should be used in their context. Before implementation, consideration also should be given to the model of care in place, including availability of adequate support to address findings from a given screening tool. Additionally, linguistic translation alone may be insufficient. Cross-cultural adaptation of these tools also is necessary to account for cultural and socioeconomic contexts. For example, the DASI tool has been developed and validated in Sinhala for use in Sri Lankan populations.^48^

A strength of this Delphi study was the involvement of 308 multidisciplinary prehabilitation experts across 49 countries. It also incorporated screening tools across three domains (physical, nutritional, psychological) identified through a comprehensive scoping review of screening tools used for patients undergoing gastrointestinal cancer surgery. Combined, this allowed expert insight via Delphi methodology regarding optimal tools based on those currently available in clinical practice.

A limitation of this study was its basis in subjective opinions of a select group of participants with self-nominated expertise. Thus, the findings do not consider the clinimetric properties of consensus tools. To ensure that screening tools effectively identify those at risk, they need to be prospectively tested in the population and setting of interest (i.e., gastrointestinal cancer surgery). To address this limitation, the next phase of this research program involves development of a self-reported, online screening tool to be assessed through an international cohort study.

Another limitation was that those who responded to the survey self-nominated as experts in prehabilitation, and the expertise of the responders was not verified. Similarly, people with lived experience (consumers), were not involved in the rating of tools. Additionally, the final cohort of respondents included a significant portion of surgeons and anesthesiologists (*n* = 198, 66%) but was underrepresented by psychologists and psychiatrists (*n* = 4, 1%), dietitians (*n* = 15, 5%), and physiotherapists (*n* = 11, 4%), which may limit the conclusions that can be drawn regarding results within assessed domains, especially when these professions likely have the greatest experience with self-report measures in clinical practice. However, in the second and third Delphi rounds of our study, experts were given the opportunity to elect the domains in which they held expertise and were invited to rate only tools within elected domains. A previous study evaluating core outcome sets for prehabilitation of those undergoing colorectal surgery had a similar sample in which surgeons and anesthesiologists were well-represented.^49^

## Conclusion

Based on a Delphi consensus of global prehabilitation experts, self-reported screening tools appropriate for use with adults undergoing gastrointestinal cancer surgery were identified across physical, nutritional, and psychological domains. Consensus was achieved for 10 screening tools. The choice of which tools to use should be based on the local context of implementation. Findings from this study will facilitate development of a multimodal self-reported online tool, which will be tested through a prospective international longitudinal cohort study aiming to identify modifiable factors allowing for preoperative interventions.

## Supplementary Information

Below is the link to the electronic supplementary material.

## Data Availability

Deidentified data files may be made available from the authors upon reasonable request, subject to permission bring granted by the Sydney Local Health District Human Research and Ethics Committee.
