# Correction: Expert Consensus on Self-Reported Physical, Nutritional, and Psychological Screening Tools for Prehabilitation in Gastrointestinal Cancer Surgery: An International Delphi Study

**DOI:** 10.1245/s10434-026-20360-6

**Published:** 2026-09-02

**Authors:** Jack Reeves, Sascha Karunaratne, Kate Alexander, Alexandria Petridis, Sharon Carey, Haryana M. Dhillon, Linda Denehy, Michael Solomon, Bernhard Riedel, Cherry Koh, Kate McBride, Chelsia Gillis, Kate White, Nicholas Hirst, Zaid Shadid, Ishraque Rahman, Neil Pillinger, Daniel Steffens, Global (P)rehabilitation Initiative Collaborative Group, Abdalla Hadhoud, Abdelatte Ghanam, Abdelilah Ghannam, Abdelmoiz Mohamed, Abdelrahim Ahmed, Abdelrahman Mohamedali, Abdirahman Osman, Abdulaleem A. Esmail, Abdulhamid Shaban, Abdulrahman Mohammed, Abdulzahra Hussain, Abdullah Al-Mehdhar, Abdullah Alyami, Achraf Drissi-El-Bouzaidi, Aeshah Alqathafi, Ahmed Ahmayda, Ahmed Elsayed Tharwat Abdelsalam, Ahmed Ibrahim MohamedMohamed, Ahmed Musbah, Ahmed Shoman, Ahmed Wahaballah, Aiman Al-Touny, Aiman Hussien, Akrem Amer, Amina Houmada, Amr Badawy, Andrea B. Maier, Andreas Denys, Andrew Davies, Angel Becerra-Bolaños, Angela Jerath, Anjum Khan Joad, Anthony Absalom, Areej Ahmed Al-Qaidani, Aras Emre Canda, Ashleigh Maske, Ashu Sara Mathai, Awab Hamid, Ayah Attallah, Ayten Saracoglu, Badr Aldeen Al-Tayar, Barbara Sinner, Basantha Kumar Rayani, Behic Girgin, Bernhard Riedel, Bisher Sawaf, Birgitte Brandstrup, Bodil Andersson, Brendan Moran, Brendon Roxburgh, Bruno Romanò, Carol Keen, Catarina Tiselius, Chelsia Gillis, Chloe Grimmett, Christian Beilstein, Cindy Yeoh, Claus Anders Bertelsen, Dario Baratti, Darren Au, David Clark, Declan Dunne, Denny Levett, Despoina Liotiri, Dimitrios Schizas, Dominique Engel, Dorian Krsul, Edoardo Virgilio, Ebrahim Ali Mohammed Amrein, Eleni Kitsou, Elharith Abduelfattah Gaber Mohammed, Elissavet Anestiadou, Emily Traer, Engy Elkoury, Enrique Alday Muñoz, Erik Nordenström, Erdinç Kamer, Eslam Elshenawy, Ewen Harrison, Fabio Ausania, Fadwa Fuad Abdulwahid Mansoor, Fatima Elmobark, Fernando Arduini, Fevzi Cengiz, Francesk Mulita, Francesco Mongelli, Francesco Pata, Frank Frizelle, Frederik Berrevoet, Gabrielle H. Van Ramshorst, Gianluca Pellino, Grainne Sheill, Gregg Nelson, Hadeer Alsadeg Mohammed, Hande Gurbuz, Hans D. De Boer, Hamid Aslami, Hamza Elhamzaoui, Hashem Abu Serhan, Haidara Bohsas, Heleen Driessens, Helena Pereira, Helmut Raab, Iacopo Cappellini, Ian Randall, Ianthe Boden, Ileana Lulic, Ilenia Merlini, Ina Ismiarti Shariffuddin, Jakub Chmelo, James Durrand, Janine Bothe, Jason Park, Jayson Moloney, Jenelle Loeliger, Jonathan Hardman, Joost Klaase, Jose Ignacio García-Sánchez, Julia Dubowitz, Julie Hallet, Junichiro Kawamura, Jyotirmay Kirtania, Kari Clifford, Kariem El-Boghdadly, Kate McBride, Khaled Abdelwahab, Khalid Osman Mohamed, Kheng-Seong Ng, Khloud Ali M. Dahan Saeed, Kimberley Bostock, Kilian Brown, Kristy-Lee Raso, Kyrillos Nassim, Kylie Hill, Kwong Weng Loh, Lara Edbrooke, Laura Lorenzon, Lewis Matthews, Linda A. Antonucci, Linda Edgar, Lissa Spencer, Louise Brennan, Magdalena Sielewicz, Magdalena Taube, Mahmoud Eissa, Majd Chaaban, Malcolm West, Malak Ghemmeid, Malek Balkhi, Manazir Athar, Marc Pocard, Marc Van De Velde, Marco Sparavigna, Marcos Gómez Ruiz, Marie-Louise Lydrup, María García Nebreda, Marta Ubre, Massimo Falconi, Michael Hii, Michael Kelly, Miguel Leon-Arellano, Moataz Maher Emara, Mohamed Al Gharyani, Mohamed Alsori, Mohamed Elbahnasawy, Mohamed Fouad Abdrabo, Mohamed Ghula, Mohamed Said, Mohamed Sherif Ali Ahmed, Mohammed Abdallah Mohammed Kheer Almotaared, Mohammed Al-Shehari, Mohammed Anass Majbar, Mohammed Izzaldin, Mohammed Omar, Mohammed Sarhan Alkhallatee, Mogahid Ahmed, Momin Hilles, Mostafa Algabri, Moyasar Awad, Msara Haider, Muhamad Kanjo, Nada Wd Badr, Nader Hanna, Nassila Krita, Natasa Kovac, Neil Pillinger, Nermina Rizvanovic, Nivedhyaa Srinivasaraghavan, Nurhilal Kiziltoprak, Orestis Ioannidis, Omar Ahmed Abdelwahab, Osman Osman, Oumayma Lahnaoui, Pankaj Roy, Pamela Klassen, Patrice Forget, Patricia Sylla, Paul Sutton, Pawel Kabata, Pawel Mroczkowski, Per J. Nilsson, Petra Bor, Peter Ihnát, Peter Noordzij, Peter Paal, Philip H. Pucher, Piotr Major, Qiang Wang, Rafat Muharam, Raquel García Álvarez, Rasha Shrayyef, Rashed Alweshah, Reham Atef Zaki Mohamed, Rhonda Farrell, Riccardo Caruso, Riyadh Ikreedeegh, Robert Arnold, Roberto Falz, Rohan Miegel, Rosalia Navarro-Perez, Ross Dolan, Safia Adem, Saleem Ahmed, Sami Sannoufa, Sandra Auld, Sara S. Elsheikh, Sarah Abdelmohsen, Sarah Braungart, Sasa Rajsic, Sawsan Ismail, Semra Demirli Atici, Seymanur Altintas Filizoglu, Shannon Philp, Sharon Carey, Shelby Yaceczko, Simone Manfredelli, Sorrel Burden, Sophie Hogan, Stefano Mancin, Styliani Apostolaki, Susana González-Suárez, Talat Abu Salem, Tania Triantafyllou, Tarik Sammour, Tim Bright, Tom Parkington, Varsha Gorey, Vasileios Mousafeiris, Varut Lohsiriwat, Victor Zaydfudim, Walter R. Frontera, Walid Ibrahim, Waled Aljbali, Wilton Van Klei, Yasemin Yıldırım, Yousef Abo Alsoud, Zakaria Belkhadir, Abdurrahman Haddud, Abubkr Elbushary, Ahmed Mohieldin Ahmed Abdalla, Alessio Giordano, Alexander Heriot, Alexander W. Phillips, Alexey Ovezov, Alfredo Annicchiarico, Algazali Jadalla, Ali Elhadidy, Alice Jay, Alison Jackson, Almu’atasim Khamees, Amanh Hussien, Amine Souadka, Amr Al Hammoud, André Torbey, Anton Burlaka, Aurélien Venara, Bashir Albakosh, Brahim El Ahamdi, Cillian Clancy, Claire Hackett, Cristina Petrisor, Daniel Santa Mina, Evgenia Mela, Hajer Alsadeg Mohammed, Ian Daniels, Julio Mayol, June Davis, Kelcie Cole, Kemal Tolga Saracoglu, Ker-Kan Tan, Khabab Abbasher Hussien Mohamed Ahmed, Liane Feldman, Mohamed Attia Elfadali, Mohammad Badr Almoshantaf, Mohammed Babikir, Moise Alida, Osama Mohamed, Padraig Buggy, Pierre-grégoire Guinot, Ragdah Mohammed, Rajeswari Alagirimunuswamy, Rand AlShrbaji, Rayan Wedatalla, Selman Sökmen, Shady Abdo Saad Ramadan, Sohaib Hasan Abdullah Ezzi, Soumia Bechka, Stephanie Castagnini, Wafaa Abdel-Elsalam

**Affiliations:** 1 Surgical Outcomes Research Centre (SOuRCe), Royal Prince Alfred Hospital, Sydney, Australia; 2 Discipline of Physiotherapy, Graduate School of Health, Faculty of Health, University of Technology Sydney, Sydney, NSW Australia; 3 Physiotherapy Department, Royal Prince Alfred Hospital, Sydney, Australia; 4 Faculty of Medicine and Health, Central Clinical School, The University of Sydney, Sydney, Australia; 5 Institute of Academic Surgery (IAS), Royal Prince Alfred Hospital, Sydney, Australia; 6 Faculty of Science, School of Psychology, Psycho-oncology Cooperative Research Group, The University of Sydney, Sydney, Australia; 7 Department of Health Services Research, Allied Health, Peter MacCallum Cancer Centre, Melbourne, Australia; 8 Department of Physiotherapy, Faculty of Medicine Dentistry and Health Sciences, The University of Melbourne, Melbourne, Australia; 9 The Sir Peter MacCallum Department of Oncology, and The Department of Critical Care, The University of Melbourne, Melbourne, Australia; 10 Department of Anaesthetics, Perioperative Medicine, and Pain Medicine, Peter MacCallum Cancer Centre, Melbourne, Australia; 11 School of Human Nutrition, McGill University, Montreal, QC Canada; 12 Susan Wakil School of Nursing, Faculty of Medicine and Health and Daffodil Centre, The University of Sydney, Sydney, Australia; 13 Department of Anaesthetics, Royal Prince Alfred Hospital, Sydney, Australia

**Correction to: Annals of Surgical Oncology (2026) 33:5751–5761** **https://doi.org/10.1245/s10434-026-19356-z**

In the original online version of this article there were collaborators missing from the Global (P)rehabilitation Initiative Collaborative Group listed in the Acknowledgments. The revised Acknowledgments is as follows:

The authors thank all members of the Global (P)rehabilitation Initiative (https://source.sydney.edu.au/global-prehabilitation-initiative/) who participated in this study. Professor Daniel Steffens is supported by a Cancer Institute NSW Career Development Fellowship. Global (P)rehabilitation Initiative Collaborative Group: Abdalla Hadhoud; Abdelatte Ghanam; Abdelilah Ghannam; Abdelmoiz Mohamed; Abdelrahim Ahmed; Abdelrahman Mohamedali; Abdirahman Osman; Abdulaleem A Esmail; Abdulhamid Shaban; Abdulrahman Mohammed; Abdulzahra Hussain; Abdullah Al-Mehdhar; Abdullah Alyami; Achraf Drissi-El-Bouzaidi; Aeshah Alqathafi; Ahmed Ahmayda; Ahmed Elsayed Tharwat Abdelsalam; Ahmed Mohamed Ibrahim Mohamed; Ahmed Musbah; Ahmed Shoman; Ahmed Wahaballah; Aiman Al-Touny; Aiman Hussien; Akrem Amer; Amina Houmada; Amr Badawy; Andrea B. Maier; Andreas Denys; Andrew Davies; Angel Becerra-Bolaños; Angela Jerath; Anjum Khan Joad; Anthony Absalom; Areej Ahmed Al-Qaidani; Aras Emre Canda; Ashleigh Maske; Ashu Sara Mathai; Awab Hamid; Ayah Attallah; Ayten Saracoglu; Badr Aldeen Al-Tayar; Barbara Sinner; Basantha Kumar Rayani; Behic Girgin; Bernhard Riedel; Bisher Sawaf; Birgitte Brandstrup; Bodil Andersson; Brendan Moran; Brendon Roxburgh; Bruno Romanò; Carol Keen; Catarina Tiselius; Chelsia Gillis; Chloe Grimmett; Christian Beilstein; Cindy Yeoh; Claus Anders Bertelsen; Dario Baratti; Darren Au; David Clark; Declan Dunne; Denny Levett; Despoina Liotiri; Dimitrios Schizas; Dominique Engel; Dorian Krsul; Edoardo Virgilio; Ebrahim Ali Mohammed Amrein; Eleni Kitsou; Elharith Abduelfattah Gaber Mohammed; Elissavet Anestiadou; Emily Traer; Engy Elkoury; Enrique Alday Muñoz; Erik Nordenström; Erdinç Kamer; Eslam Elshenawy; Ewen Harrison; Fabio Ausania; Fadwa Fuad Abdulwahid Mansoor; Fatima Elmobark; Fernando Arduini; Fevzi Cengiz; Francesk Mulita; Francesco Mongelli; Francesco Pata; Frank Frizelle; Frederik Berrevoet; Gabrielle H. Van Ramshorst; Gianluca Pellino; Grainne Sheill; Gregg Nelson; Hadeer Alsadeg Mohammed; Hande Gurbuz; Hans D De Boer; Hamid Aslami; Hamza Elhamzaoui; Hashem Abu Serhan; Haidara Bohsas; Heleen Driessens; Helena Pereira; Helmut Raab; Iacopo Cappellini; Ian Randall; Ianthe Boden; Ileana Lulic; Ilenia Merlini; Ina Ismiarti Shariffuddin; Jakub Chmelo; James Durrand; Janine Bothe; Jason Park; Jayson Moloney; Jenelle Loeliger; Jonathan Hardman; Joost Klaase; Jose Ignacio García-Sánchez; Julia Dubowitz; Julie Hallet; Junichiro Kawamura; Jyotirmay Kirtania; Kari Clifford; Kariem El-Boghdadly; Kate McBride; Khaled Abdelwahab; Khalid Osman Mohamed; Kheng-Seong Ng; Khloud Ali M. Dahan Saeed; Kimberley Bostock; Kilian Brown; Kristy-Lee Raso; Kyrillos Nassim; Kylie Hill; Kwong Weng Loh; Lara Edbrooke; Laura Lorenzon; Lewis Matthews; Linda A. Antonucci; Linda Edgar; Lissa Spencer; Louise Brennan; Magdalena Sielewicz; Magdalena Taube; Mahmoud Eissa; Majd Chaaban; Malcolm West; Malak Ghemmeid; Malek Balkhi; Manazir Athar; Marc Pocard; Marc Van De Velde; Marco Sparavigna; Marcos Gómez Ruiz; Marie-Louise Lydrup; María García Nebreda; Marta Ubre; Massimo Falconi; Michael Hii; Michael Kelly; Miguel Leon-Arellano; Moataz Maher Emara; Mohamed Al Gharyani; Mohamed Alsori; Mohamed Elbahnasawy; Mohamed Fouad Abdrabo; Mohamed Ghula; Mohamed Said; Mohamed Sherif Ali Ahmed; Mohammed Abdallah Mohammed Kheer Almotaared; Mohammed Al-Shehari; Mohammed Anass Majbar; Mohammed Izzaldin; Mohammed Omar; Mohammed Sarhan Alkhallatee; Mogahid Ahmed; Momin Hilles; Mostafa Algabri; Moyasar Awad; Msara Haider; Muhamad Kanjo; Nada Wd Badr; Nader Hanna; Nassila Krita; Natasa Kovac; Neil Pillinger; Nermina Rizvanovic; Nivedhyaa Srinivasaraghavan; Nurhilal Kiziltoprak; Orestis Ioannidis; Omar Ahmed Abdelwahab; Osman Osman; Oumayma Lahnaoui; Pankaj Roy; Pamela Klassen; Patrice Forget; Patricia Sylla; Paul Sutton; Pawel Kabata; Pawel Mroczkowski; Per J Nilsson; Petra Bor; Peter Ihnát; Peter Noordzij; Peter Paal; Philip H Pucher; Piotr Major; Qiang Wang; Rafat Muharam; Raquel García Álvarez; Rasha Shrayyef; Rashed Alweshah; Reham Atef Zaki Mohamed; Rhonda Farrell; Riccardo Caruso; Riyadh Ikreedeegh; Robert Arnold; Roberto Falz; Rohan Miegel; Rosalia Navarro-Perez; Ross Dolan; Safia Adem; Saleem Ahmed; Sami Sannoufa; Sandra Auld; Sara S. Elsheikh; Sarah Abdelmohsen; Sarah Braungart; Sasa Rajsic; Sawsan Ismail; Semra Demirli Atici; Seymanur Altintas Filizoglu; Shannon Philp; Sharon Carey; Shelby Yaceczko; Simone Manfredelli; Sorrel Burden; Sophie Hogan; Stefano Mancin; Styliani Apostolaki; Susana González-Suárez; Talat Abu Salem; Tania Triantafyllou; Tarik Sammour; Tim Bright; Tom Parkington; Varsha Gorey; Vasileios Mousafeiris; Varut Lohsiriwat; Victor Zaydfudim; Walter R. Frontera; Walid Ibrahim; Waled Aljbali; Wilton Van Klei; Yasemin Yıldırım; Yousef Abo Alsoud; Zakaria Belkhadir; Abdurrahman Haddud; Abubkr Elbushary; Ahmed Mohieldin Ahmed Abdalla; Alessio Giordano; Alexander Heriot; Alexander W. Phillips; Alexey Ovezov; Alfredo Annicchiarico; Algazali Jadalla; Ali Elhadidy; Alice Jay; Alison Jackson; Almu'atasim Khamees; Amanh Hussien; Amine Souadka; Amr Al Hammoud; André Torbey; Anton Burlaka; Aurélien Venara; Bashir Albakosh; Brahim El Ahamdi; Cillian Clancy; Claire Hackett; Cristina Petrisor; Daniel Santa Mina; Evgenia Mela; Hajer Alsadeg Mohammed; Ian Daniels; Julio Mayol; June Davis; Kelcie Cole; Kemal Tolga Saracoglu; Ker-Kan Tan; Khabab Abbasher Hussien Mohamed Ahmed; Liane Feldman; Mohamed Attia Elfadali; Mohammad Badr Almoshantaf; Mohammed Babikir; Moise Alida; Osama Mohamed; Padraig Buggy; Pierre-grégoire Guinot; Ragdah Mohammed; Rajeswari Alagirimunuswamy; Rand AlShrbaji; Rayan Wedatalla; Selman Sökmen; Shady Abdo Saad Ramadan; Sohaib Hasan Abdullah Ezzi; Soumia Bechka; Stephanie Castagnini; Wafaa Abdel-Elsalam.

The original article was corrected.

