# Supplementary material for: Expert Consensus on Self-Reported Physical, Nutritional, and Psychological Screening Tools for Prehabilitation in Gastrointestinal Cancer Surgery: An International Delphi Study
Source: Ann Surg Oncol. 2026 Mar 14;33(6):5751–61. doi: 10.1245/s10434-026-19356-z (PMC13179180; doi:10.1245/s10434-026-19356-z)
Supplement: Supplementary file 1 — Supplementary file1 (DOCX 17 KB) [file 10434_2026_19356_MOESM1_ESM.docx]

| **Welcome to the first round survey.** |
| --- |
| Thank you for participating in this important research to establish consensus on the most appropriate patient-reported screening tools for gastrointestinal cancer surgery patients. **What This Study Involves**: We are seeking expert consensus to identify the best **self-reported screening tools** that evaluate **physical**, **nutritional**, and **psychological** health before **gastrointestinal cancer surgery**. The selected tools will be implemented in subsequent international research to risk-stratify patients and identify those at higher risk of poor postoperative outcomes. This consensus is crucial as it will directly inform targeted preoperative interventions to improve patient care. **Study Process**: This three-round Delphi survey presents screening tools identified through our comprehensive scoping review. You will rate each tool's appropriateness using a 5-point scale and have the opportunity to suggest additional relevant tools we may have missed. Through iterative rounds, we will achieve consensus on the most suitable patient-reported screening tools. **Inclusion Criteria**: Only patient-reported tools requiring no clinician assessment, physical examination, or biochemical markers are included. Quality-of-life tools and geriatric-specific assessments are excluded from this study. Tools intended for geriatric populations yet which are applicable to general populations can be included. **Your Expert Input**: Your professional expertise is vital in selecting tools that will ultimately help identify high-risk patients and guide preoperative interventions to improve surgical outcomes for gastrointestinal cancer patients worldwide.  The complete study protocol is attached below and available at: https://www.mdpi.com/2072-6694/17/5/861  Please rate the following screening tools using the 5-point scale provided (1 = no importance, 2 = limited importance, 3 = neither important nor unimportant, 4 = somewhat important, 5 = critically important):  Attachment: [An Online Preoperative Screening Tool to Optimize Care for Patients Undergoing Cancer Surgery - A Mixed-Method Study Protocol.pdf](https://redcap.slhd.nsw.gov.au/redcap_v15.5.18/DataEntry/file_download.php?pid=8383&page=first_round_survey&type=attachment&field_name=describe&hidden_edit=1&record=348&event_id=20917&doc_id_hash=273d0540b1c50f217593a49f4a7d60661cb3f710c67b99ebd1db23495f09ed9d52073ba80df0d88f7131c8d1c8dd2bc4e765788c556f961032a55f9f6ff5b85b&instance=1&id=394325)(0.65 MB) |
| **Please rate the following screening tools using the 5-point scale provided (1 = no importance, 2 = limited importance, 3 = neither important nor unimportant, 4 = somewhat important, 5 = critically important).  Please differentiate between tools by assigning varied scores rather than rating all tools identically - reserve higher scores for tools you consider most valuable and lower scores for those you find less suitable. While you may assign similar scores to tools of comparable value, please avoid giving the exact same rating to all tools.** |

Example of information provided on a screening tool (which includes an attachment to a PDF copy of the tool).

| Duke Activity Status Index (DASI) | |
| --- | --- |
| Tool Description | The Duke Activity Status Index (DASI) is a validated, self-administered questionnaire designed to assess the functional capacity of adults, particularly those with cardiovascular conditions. It evaluates an individual's ability to perform daily activities, ranging from basic self-care to strenuous physical tasks. The questionnaire provides an estimate of a patient's peak oxygen uptake (VO₂ max) and metabolic equivalents (METs). |
| Recall Period | Current functional ability. |
| Number of Items | 12 items |
| Response Format | Yes/no checklist. |
| Time to complete | 5 mins |
| Scoring | Each "Yes" response is weighted; total score is the sum of item weights which can be used to estimate peak VO₂. |
